# The inhibition of human farnesyl pyrophosphate synthase by nitrogen-containing bisphosphonates. Elucidating the role of active site threonine 201 and tyrosine 204 residues using enzyme mutants^☆^

**DOI:** 10.1016/j.bone.2015.08.020

**Published:** 2015-12

**Authors:** Maria K. Tsoumpra, Joao R. Muniz, Bobby L. Barnett, Aaron A. Kwaasi, Ewa S. Pilka, Kathryn L. Kavanagh, Artem Evdokimov, Richard L. Walter, Frank Von Delft, Frank H. Ebetino, Udo Oppermann, R. Graham G. Russell, James E. Dunford

**Affiliations:** a Botnar Research Centre, Nuffield Department of Orthopaedics Rheumatology and Musculoskeletal Science, University of Oxford, Oxford OX3 7LD, UK; b Structural Genomics Consortium, University of Oxford, Oxford OX3 7DQ, UK; c Chemistry Department, University of Cincinnati, Cincinnati, OH 45221, USA; d Department of Chemistry, University of Rochester, Rochester, NY 14627, USA; e Mellanby Centre for Bone Research, University of Sheffield Medical School, Sheffield S10 2RX, UK; f Monsanto, St Louis, MO, USA.; g Shamrock Structures, Woodridge, Illinois, USA.

**Keywords:** DMAPP, dimethylallyl pyrophosphate, FPP, farnesyl pyrophosphate, FPPS, farnesyl pyrophosphate synthase, GPP, geranyl pyrophosphate, IPP, isopentenyl pyrophosphate, N-BP, nitrogen-containing bisphosphonates, TCEP, tris(2-carboxyethyl)phosphine, RIS, Risedronate, ZOL, Zoledronate, PAM, Pamidronate, ALN, Alendronate, IBN, Ibandronate, Bisphosphonate, Farnesyl pyrophosphate synthase, Inhibition mechanism, Drug binding, Substrate binding, Active site mutant

## Abstract

Farnesyl pyrophosphate synthase (FPPS) is the major molecular target of nitrogen-containing bisphosphonates (N-BPs), used clinically as bone resorption inhibitors. We investigated the role of threonine 201 (Thr201) and tyrosine 204 (Tyr204) residues in substrate binding, catalysis and inhibition by N-BPs, employing kinetic and crystallographic studies of mutated FPPS proteins.

Mutants of Thr201 illustrated the importance of the methyl group in aiding the formation of the Isopentenyl pyrophosphate (IPP) binding site, while Tyr204 mutations revealed the unknown role of this residue in both catalysis and IPP binding. The interaction between Thr201 and the side chain nitrogen of N-BP was shown to be important for tight binding inhibition by zoledronate (ZOL) and risedronate (RIS), although RIS was also still capable of interacting with the main-chain carbonyl of Lys200. The interaction of RIS with the phenyl ring of Tyr204 proved essential for the maintenance of the isomerized enzyme-inhibitor complex. Studies with conformationally restricted analogues of RIS reaffirmed the importance of Thr201 in the formation of hydrogen bonds with N-BPs.

In conclusion we have identified new features of FPPS inhibition by N-BPs and revealed unknown roles of the active site residues in catalysis and substrate binding.

## Highlights

• Bisphosphonate side chain nitrogen interaction with side chain of Thr201 is essential for maximal inhibition by ZOL
• Tyr204 is essential for maximal inhibition by RIS but not aminoalkyl bisphosphonates
• Active site residues Thr201 and Tyr204 aid formation of the second substrate IPP binding site

## 1 Introduction

Farnesyl pyrophosphate synthase (FPPS) is a key branch point enzyme in the mevalonate pathway, the exclusive route of isoprenoid production in animals, involved in cholesterol biosynthesis and synthesis of intermediates important for intracellular signalling and growth control [1]. Inhibition of FPPS causes a reduction in farnesyl pyrophosphate (FPP) and geranylgeranylpyrophosphate (GGPP) levels leading to impaired prenylation and defective intracellular localization of GTPase signalling proteins such as Ras, Rho and Rac [2], [3]. These proteins are essential for osteoclast cell processes such as bone resorption, cell movement, cytoskeletal rearrangement and apoptosis [4], [5]. FPPS has been confirmed as the primary molecular target of the nitrogen-containing bisphosphonates (N-BPs) that are potent inhibitors of osteoclastic activity [6]. Several members of this class are important clinically used drugs for conditions characterized by excessive bone resorption such as Paget's disease [7], multiple myeloma [8], bone metastases [9] and osteoporosis [10], [11].

FPPS is a homodimeric enzyme, made up of two 42 kDa monomers, that initially catalyse a head-to-tail condensation of the 5-carbon allylic substrate dimethylallyl pyrophosphate (DMAPP) with isopentenyl pyrophosphate (IPP) to yield the C_10_ geranylpyrophosphate (GPP). Subsequently, a second head-to-tail condensation of GPP and IPP within this enzyme yields the C_15_ farnesyl pyrophosphate (FPP). The reaction is thought to proceed by a highly ordered three step ionization-condensation-elimination mechanism, via the formation of a carbocation intermediate, which is stabilized by the presence of the OH group of Thr201, the main-chain carbonyl oxygen of Lys200 and the side chain oxygen of glutamine 240 [12], [13], [14]. The ionization of DMAPP is facilitated by the enzyme-bound tri-nuclear Mg^2 +^ cluster where all three Mg^2 +^ ions form salt-bridges with the un-esterified oxygens of the pyrophosphate, enhancing the juxtaposition of the hydrophobic C_5_ isoprenoid tail of the IPP into the correct conformation for subsequent catalysis [12], [15]. The carbocation formed condenses with the nucleophilic C_3_–C_5_ double bond of IPP. Finally, elimination of the isoprenoid reaction product, GPP, is achieved by de-protonation of the condensed intermediate by the free pyrophosphate oxygen [12].

The structures of human FPPS co-crystallised with several clinically utilised N-BPs demonstrated that N-BPs occupy the “allylic substrate binding pocket” (DMAPP/GPP) of FPPS, in agreement with the proposed kinetic model [16], [17]. Coordination of the phosphonate groups of N-BP is facilitated via interactions of Mg^2 +^ ions with the same aspartate-rich motifs of FPPS that bind the pyrophosphate moieties of the allylic substrate [16]. In the case of two of the most potent inhibitors of FPPS, risedronate (RIS) and zoledronate (ZOL), the N-BP binding is strengthened by hydrogen bond interactions of the protonated nitrogen atom within the heterocyclic ring of the side chain of the N-BP with the conserved main-chain carbonyl oxygen of Lys200 and the hydroxyl group of the Thr201 side chain, mimicking the stabilization of a carbocation intermediate [12], [16], [17], [18]. In the case of pamidronate (PAM), the position of the hydroxyl of the Tyr204 is found close to the amino-group in the alkyl side chain of PAM (3 Å) and, favours formation of a hydrogen bond thus accounting for the intermediate to weak enzyme inhibitory effect of PAM [17]. The N-BP:FPPS interactions are complex in nature due to N-BPs having two different modes of inhibition: a rapid and reversible competitive inhibition with regard to the allylic substrate DMAPP/GPP, reflecting the inhibition constant Ki, and an uncompetitive or mixed-type inhibition in relation to IPP [16], [19]. The latter is a time-dependent inhibition, arising due to an enzyme isomerization occurring in two stages: i) N-BP binds to the allylic site and forms the IPP binding site and ii) IPP binds to the FPPS:N-BP complex and closes the active site using amino acid residues found in the C-terminus of FPPS (Lys350, Arg351, Arg352, Lys353) [16], [17]. This complex inhibition mechanism is expressed as a final isomerized inhibition constant Ki*. The tendency of the enzyme to remain in the isomerized state is given by the isomerization constant, K_isom_ = (Ki − Ki*) / Ki*.

The biological effects of the bisphosphonates are still being extensively studied and many new applications are emerging, including improvements to current therapies [20], [21]. There is also interest in mevalonate pathway modulation as a potential target for the treatment of diseases caused by protozoan parasites, such as toxoplasmosis [22], leishmaniasis [23], [24], Chagas disease [25], [26] and malaria [27], [28]. An understanding of the molecular interaction of pivotal FPPS amino-acid residues with the side chain of selected BPs (Table 1) may enable the synthesis of additional N-BP analogues that will selectively target the FPPS enzymes in humans and other species. For the applications focused more on non-skeletal tissue targets, developments in the design and measurements of lower bone affinity bisphosphonates have evolved that may advance the utility of the class, both with regards to lower skeletal uptake and enhanced activity [6].

Having initially identified the importance of the conserved Thr201 and Tyr204 as two pivotal active site residues contributing to the inhibitory action of the more potent N-BPs we have sought to define their roles in greater detail. This has been enabled by the generation of the threonine-to-serine (T201S) and tyrosine-to-phenylalanine (Y204F) mutants, which we hypothesised would not disrupt the proposed Thr201:N-BP hydrogen bond and hydrophobic interactions respectively whereas a Thr201 to alanine (T201A) and Tyr204 to alanine (Y204A) mutant should abolish such interactions.

## 2 Methods

Oligonucleotides were purchased from Eurofins MWG. Modified standard Expression LIC vectors were all based on pET-28a (Novagen, Madison, USA) and were provided by Structural Genomics Consortium, Oxford, UK. Unlabelled geranyl pyrophosphate (GPP) and isopentenyl pyrophosphate (IPP) were purchased from Isoprenoids LC (Tampa FI, USA). Isopentenyl Pyrophosphate (1-^14^C) triammonium salt was purchased from American Radiochem Corp UK Ltd (Cardiff, UK). All BPs were obtained from Procter and Gamble Pharmaceuticals (Cincinnati, USA), except PAM and ALN which were from Sigma.

### 2.1 Manufacture of mutants

Generation of FPPS mutants was carried out with QuickChange Site-directed mutagenesis kits from Agilent Technologies used according to manufacturer's instructions. Mutations were confirmed by DNA sequencing analysis (LARK Technologies, Takeley, UK) and by ESI-TOF mass spectrometry (Agilent).

### 2.2 Expression and purification of recombinant human FPPS

FPPS was expressed as described previously [16].

### 2.3 FPPS synthase assay

FPPS activity was measured as described previously [16].

### 2.4 Kinetic models and calculations of constants

Data were fitted to the appropriate kinetic models by nonlinear regression analysis using Graphpad Prism®. Determination of tight-binding ligand characteristics were analysed based on the equation developed by Morrison [29].

$$\frac{Vi}{Vo}=1-\frac{\left(\left[E\right]+\left[I\right]+\mathrm{Kiapp}\right)-\sqrt{{\left(\left[E\right]+\left[I\right]+\mathrm{Kiapp}\right)}_{2}-4\left[E\right]\left[I\right]}}{2\left[E\right]}$$

$$\frac{Vi}{Vo}=1-\frac{\left(\left[E\right]+\left[I\right]+K_{i}^{*\mathrm{app}}\right)-\sqrt{{\left(\left[E\right]+\left[I\right]+K_{i}^{*\mathrm{app}}\right)}^{2}-4\left[E\right]\left[I\right]}}{2\left[E\right]}$$

Initial inhibition constant (*K*_i_), overall inhibition constant (K_i_*) and isomerisation constant (K_isom_) values were calculated as previously described [19].

### 2.5 Crystallization and data collection of human FPPS with N-BPs

Tobacco etch virus protease-cleaved FPPS was concentrated up to 15 mg/ml. N-BPs were prepared as a 20 mM stock solution in 100 mM Tris–HCl pH 7.7 and were added to a final concentration of 2 mM. MgCl_2_ was prepared as a 100 mM aqueous stock solution and added to a final concentration of 4 mM. Crystals were grown by vapour diffusion at 20 °C in 300 nl sitting drops by mixing 200 nl of protein solution with 100 nl of precipitant. For the 4Q23 structure the precipitant was 2 M (NH_4_)_2_SO_4_, 0.1 M Acetate, pH 4.6 for all other structures the precipitant consisted of 0.2 M NH_4_Cl, 20% (w/v) PEG 6000, 10% ethylene glycol, pH 7.5. A single crystal was transferred to cryoprotectant solution composed of 20% (v/v) ethylene glycol and 80% well solution and flash cooled in liquid nitrogen.

### 2.6 Data processing and refinement

Indexing and integration of collected data was performed using MOSFLM [30] and symmetry-related reflections were scaled by SCALA [31] and converted into amplitudes by TRUNCATE. Initial phases for the FPPS:N-BP complexes were calculated by molecular replacement implemented in PHASER [32] using the wtFPPS FPPS in complex with ZOL (PDB code: 1ZW5) as a starting model. Iterative rounds of model building in COOT [33] and refinement using REFMAC5 [34] resulted in the final models. All refined structures have been deposited into the Protein Data Bank (Table 6).

## 3 Statistical analysis

K_i_, K_i_* and K_isom_ were analysed for significance using one way Anova with a Tukey's post hoc test and also by Students T-Test. Inhibition kinetic curves were further differentiated by comparing best fit values for IC_50_ to selected data sets using an F-test. All statistical analysis was performed with Graphpad Prism^®^

## 4 Results

### 4.1 Kinetic data for T201S, T201A, Y204F, Y204A

Determination of kinetic parameters for all mutants (Table 2) revealed a K_m_ for the allylic GPP substrate comparable to that of the wtFPPS (Tukey post-hoc test, p > 0.5), with the exception of T201S FPPS where the K_m_^GPP^ was slightly lower (p < 0.01). The K_m_^IPP^ was significantly increased in all mutants with Y204A showing the greatest (24-fold) increase. Consequently, the catalytic efficiency (k_cat_/K_m_) for the Thr201 mutants was unaffected with respect to GPP but decreased for the other mutants, whereas all the other mutants showed reduced catalytic efficiency with regard to IPP.

### 4.2 The pH activity profile of Tyr204 FPPS mutants

**Fig. 1:**
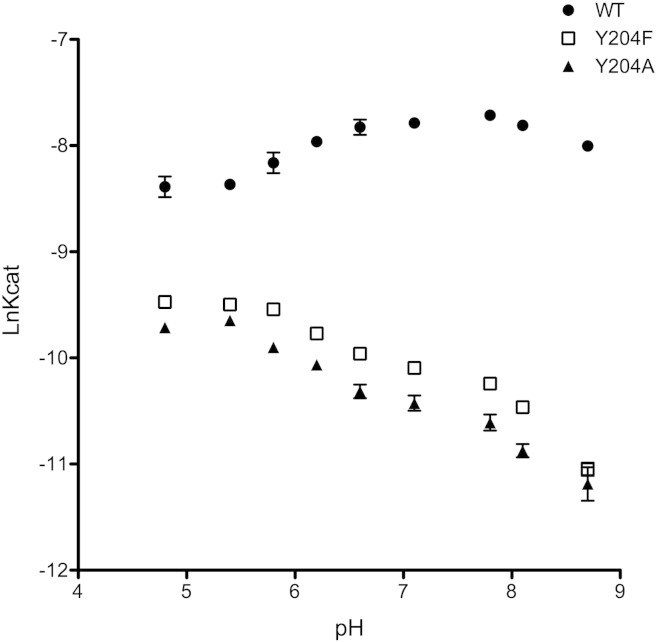
Optimal catalytic activity of wtFPPS and Tyr204 FPPS mutants is observed in different pH environment. The catalytic profile of wtFPPS is a bell shape curve with optimal catalysis observed at pH values between 6 to 8. Optimal catalysis in Y204F and Y204A FPPS mutants is observed in an acidic environment. Graph represents means obtained from four individual experiments.

The side chain of Tyr204 has a hydroxyl moiety which could conceivably act as a proton donor during catalysis. This ability will vary with the pH of the surrounding solvent. To investigate this possible role the catalytic activity for wtFPPS and Tyr204 mutated FPPS constructs was evaluated at nine different pH conditions (Fig. 1). The pH profile obtained for wtFPPS is a typical bell shape curve, where catalytic activity is optimal at pH values ranging from 6 to 8 and is reduced at either high or low pH. This pattern indicates the presence of two ionizing groups taking part in catalysis, expressed by two different pKa values calculated as 5.3 and 9.3. This is in striking contrast to both of the Tyr204 mutants which display their highest catalytic turnover in an acidic environment. The reduction of catalytic activity in the Tyr204 mutants supports the suppression of an ionization mechanism involving the hydroxyl of Tyr204. The elevated enzymatic activity at low pH appears to compensate for the loss of ionization/proton donation by the absent hydroxyl moiety.

### 4.3 Examination of the IPP binding site in the FPPS mutants

**Fig. 2:**
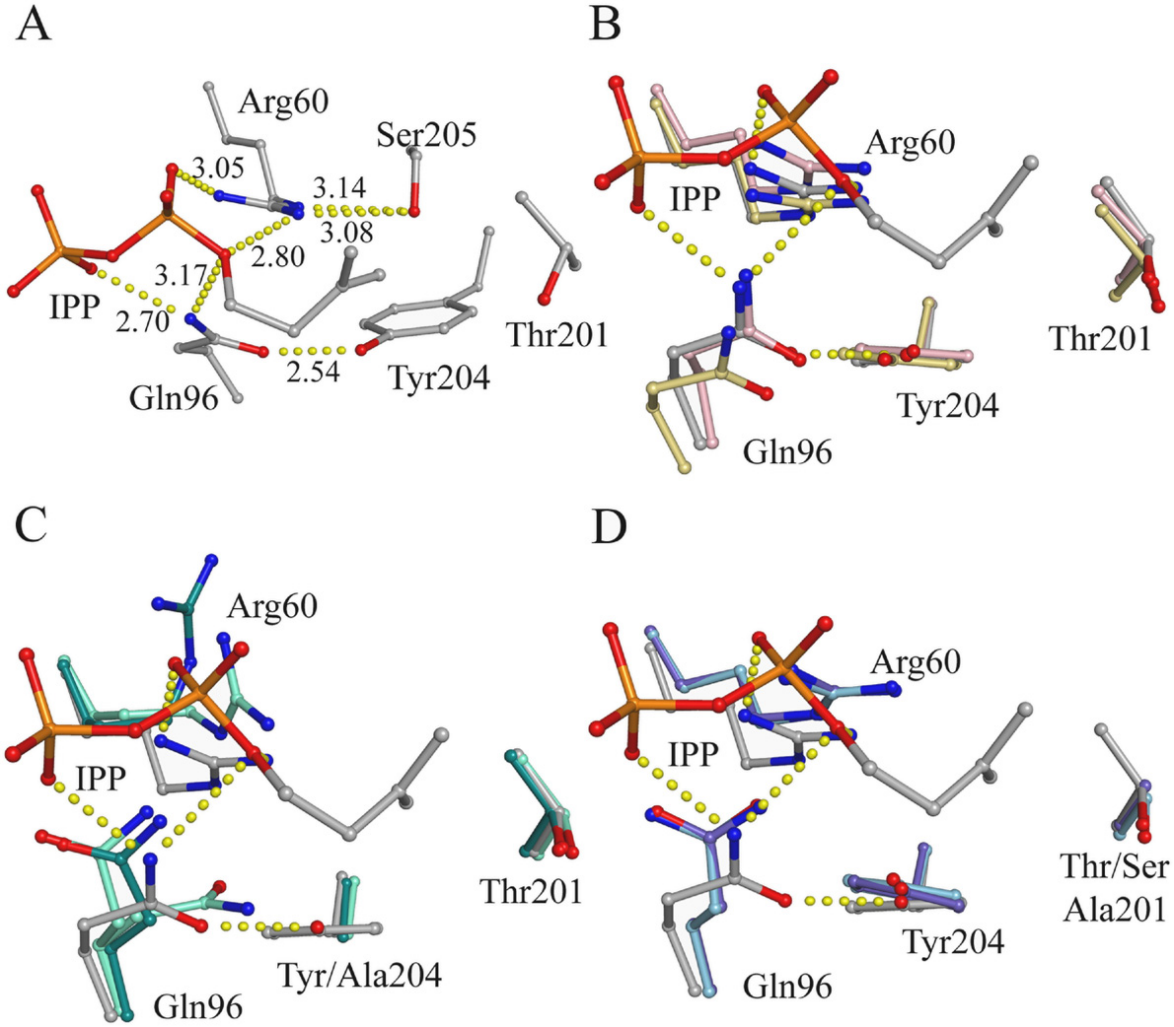
Interactions in the IPP binding site. Hydrogen-bonding interactions for Gln96 and Arg60 are shown for the WT + ZOL + IPP structure (grey carbons, 1ZW5) as dashed yellow dotted lines. A) Gln96 and Arg60 electrostatic interactions with IPP, Tyr204 and Ser205 as seen in WT + ZOL + IPP. Distances for H-bonds are given in Å. B) Overlay of apo wtFPPS (yellow, 2F7M), WT + RIS (pink, 1YV5) and WT + ZOL + IPP FPPS crystal structures showing conserved orientation of Gln96 and Arg60. C) In FPPS tyrosine mutants Y204A + ZOL (bluegreen, 4KQ5) and Y204A + PAM (light green, 4KPJ) Gln96 adopts an extended conformation that pushes Arg60 away from its optimal IPP binding position. D) In Thr201 FPPS mutants, T201A + ZOL (purple, 4KFA) and T201S + RIS (light blue, 2QIS), Gln96 and Arg60 adopt similar conformations as described for Tyr204 mutants.

To investigate the reduction in IPP binding affinity for the FPPS mutants, we compared structures of wtFPPS and mutant FPPS. In all the crystallographic models of FPPS:BP:IPP ternary complexes, IPP is held in place by hydrogen-bonding and salt-bridge interactions with Gln96 and Arg60. In addition, Gln96 is held in place by a hydrogen bond with Tyr204 and Arg60 is positioned by a hydrogen bond with Ser205 (Fig. 2A). A comparison of apo, binary and ternary wtFPPS structures shows that both Gln96 and Arg60 are in optimal positions for IPP binding whether ligands are present or not (Fig. 2B). Disruption of the Gln96-Tyr204 hydrogen bond, as occurs in the Tyr204 mutants, allows Gln96 to adopt a more extended conformation, observed in our Y204A mutant binary complexes (4KQ5, 4KPJ). This extended Gln96 conformation also pushes Arg60 away from its optimal position for IPP binding (Fig. 2C). As a result of these conformational changes both Gln96 and Arg60 are forced to readjust position when IPP binds to the mutant enzymes. Despite the presence of the Tyr204 hydroxyl this extended Gln96 conformation was also observed in Thr201 mutants (2QIS, 4KFA) (Fig. 2D) and is possibly due to more flexibility in the active site in the Thr201 mutants compared to wtFPPS. The hydroxyl moiety's of both residues play a role in formation of the IPP binding site in the wtFPPS. Consequently, IPP must induce changes in the mutant enzyme architecture for productive IPP binding to occur, while the active site is already favourable for IPP binding in wtFPPS. The need for the Gln96 and Arg60 side chains to move into position for productive IPP binding likely explains the increase in K_m_^IPP^ observed for the mutant enzymes.

### 4.4 The role of the methyl group of Thr201 in RIS binding

**Fig. 3:**
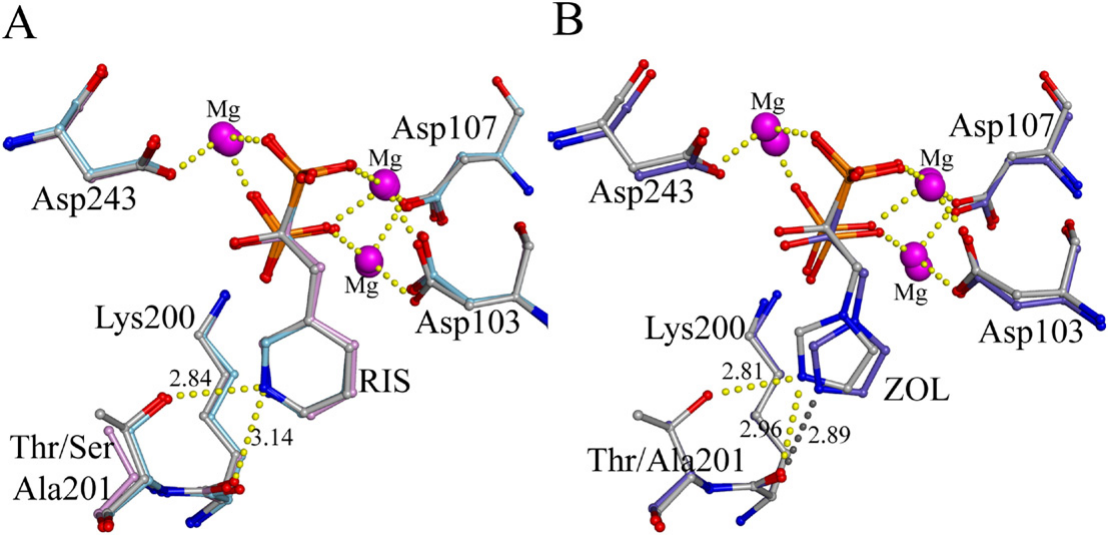
Structures of Thr201 mutants with N-BPs. A) Overlay of WT + RIS (grey, 1YV5), T201S + RIS (light blue, 2QIS) and T201A + RIS (plum, 4Q23). H-bonds are shown as yellow dashed lines and distances (Å) are given for RIS in the T201S structure. B) T201A + ZOL (purple, 4KFA) superimposed onto WT + ZOL + IPP (grey, 1ZW5). H-bond distances (Å) for the ZOL nitrogen are shown in yellow for wtFPPS and grey for T201A.

The conservative T201S mutation, which was expected to have little effect on N-BP inhibition, significantly increased both the initial competitive inhibition and also the final isomerized inhibition with a reduction in Ki and Ki*(p < 0.01) of RIS (Table 3). The crystal structure of T201S with RIS (2QIS) revealed no alteration in the positioning of RIS in the allylic binding pocket and confirmed the maintenance of the hydrogen bond network between the carbonyl of Lys200 and hydroxyl of Ser201 with the nitrogen moiety of RIS (Fig. 3A). Similarly, the position of Mg^2 +^ ions and the aspartate motifs (Asp103, Asp107 and Asp243) remained unaffected by the mutation. The RIS position did not shift significantly in the binding pocket and it is unclear which interactions contribute to the observed increased inhibition. Interestingly, the T201A mutation, which was predicted to have reduced inhibition by RIS, had no effect on Ki but showed a small increase in overall inhibition (i.e. a small decrease in Ki*). Disruption of the Thr201:RIS hydrogen bond in the T201A FPPS did not affect the orientation of the heterocyclic ring of RIS or the relative positions of key amino acids in the allylic binding pocket.

### 4.5 Kinetic studies of T201A with RIS analogues

The role of Thr201:N-BP hydrogen bond in enzyme inhibition was further investigated via kinetic studies with RIS analogues NE58018, NE58022, 8, 9 (Table 1). The lack of the nitrogen atom in NE58022 renders the analogue incapable of forming the hydrogen bond network with Lys200 and Thr201 and, as expected, the T201A mutation had no effect on the inhibition profile of NE58022 (Table 4). Because of the altered position of the nitrogen in NE58018, this analogue is less capable of forming a hydrogen bond between the heterocyclic ring and the hydroxyl moiety of Thr201 and thus becomes a weaker inhibitor of FPPS compared to RIS [19]. The inhibition of T201A FPPS by NE58018 was decreased by approximately 3-fold in the T201A mutant (Table 4) reducing the K_isom_.

In order to further investigate the importance of the interaction of the bisphosphonate nitrogen with Thr201 a conformationally restricted pair of analogues of RIS, NE58025 1R6S and NE58025 1S6R [6] were used for inhibition studies and crystallography. The double ring structure of the analogues means that compound NE58025 1R6S can only bind the enzyme with the side chain nitrogen in a similar position to that of RIS and should be able to form the hydrogen bond with Thr201. Compound NE58025 1S6R however can only bind FPPS with the side chain nitrogen away from the Thr201 with the prediction this will be the weaker inhibitor. In accordance with our predictions, NE58025 1R6S was the more potent inhibitor of the pair and even though NE58025 1R6S becomes a stronger competitive inhibitor in the initial stage of inhibition with T201A, the overall inhibition was reduced in the T201A mutant with an 8-fold increase in K_i_* and approximately 30-fold reduction in the K_isom_ relative to wtFPPS. Compound NE58025 1S6R was a much weaker inhibitor and overall inhibition was virtually unaffected by the T201A replacement (Table 4).

### 4.6 The inhibition of Tyr204 mutants by RIS

**Fig. 4:**
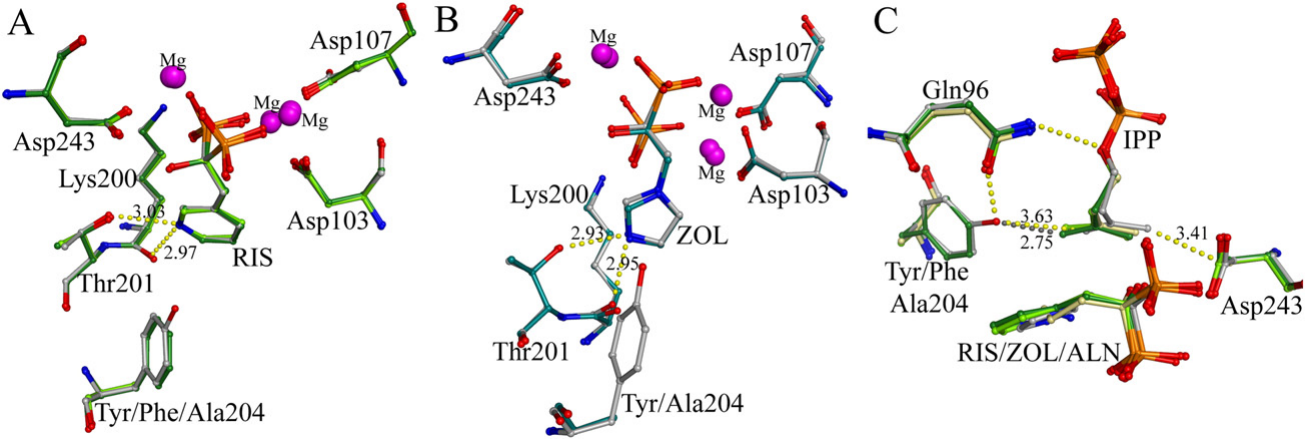
Structures of Tyr204 mutants with bound inhibitor. A) Overlay of WT + RIS (grey, 1YV5), Y204F + RIS + IPP (darkgreen, 4KPD) and Y204A + RIS + IPP (lightgreen, 4KQS). H-bond distances (Å) for the RIS nitrogen are given for wtFPPS and RIS position is unaffected by the Y204 mutation. B) Y204A + ZOL (bluegreen, 4KQ5) superimposed onto WT + ZOL + IPP (grey, 1ZW5). H-bond distances (Å) for the ZOL nitrogen are given for Y204A. C) Superimposition of WT + ZOL + IPP (grey, 1ZW5), Y204F + RIS + IPP (darkgreen, 4KPD), Y204A + RIS + IPP (lightgreen, 4KQS) and Y204A + ALN + IPP (yellow, 4KQU). In wtFPPS the IPP is sterically constrained between Asp243 and the Tyr204 hydroxyl (distances (Å) shown as dashed yellow lines). In Y204A and Y204F, lack of the hydroxyl allows the IPP C4 to shift ~ 0.9 Å into a more hydrophobic pocket. Distance from the IPP C4 in Y204F + RIS + IPP to the position where the Tyr hydroxyl would be is shown in grey.

In the Tyr204 FPPS mutants the competitive inhibition by RIS was virtually unaffected by the Y204F substitution yielding a Ki comparable to that of WT, but Ki was significantly reduced in the Y204A mutant (p < 0.001) (Table 3). However, the Ki* is significantly increased in both Y204A FPPS (p < 0.05) and Y204F FPPS. Alterations in the IPP binding site described above did not affect the position of RIS in the binding pocket in either mutant (Fig. 4A). We postulate that changes in electrostatic interactions between RIS and the mutated residue contributed to the decreased final inhibition as well as an increase in the reversibility of the enzyme isomerisation, as shown by the reduced isomerisation constant K_isom_. These results confirm that the presence of the aromatic ring and the hydroxyl moiety of Tyr204 do not play a role in the initial binding of RIS to the allylic site of FPPS, but are essential for isomerisation of the enzyme to its final closed conformation.

### 4.7 Inhibition of Thr201 and Tyr204 mutants by ZOL

ZOL is shown to form a hydrogen bond with the hydroxyl of Thr201 via the nitrogen in the side chain (1ZW5, 2F8Z) and it was predicted that the replacement of this residue with an Ala would disrupt this bonding and reduce the overall inhibition of the enzyme. The replacement of Thr201 by Ala resulted in a significant increase in Ki* and reduction in K_isom_ (p < 0.001) by ZOL but does not have any impact on the initial Ki value (Table 3). The replacement of Thr201 by a Ser residue should rescue the loss of inhibition, and indeed this was the case (Table 3). The lack of the Thr201: ZOL hydrogen bond in the T201A FPPS structure (4KFA) does not greatly alter the position of the imidazole ring in the allylic binding pocket, as it is still properly oriented by the Lys200:ZOL hydrogen bond (Fig. 3B).

The Y204F FPPS mutant exhibited no alteration in Ki for ZOL with respect to the wtFPPS (Table 3). However the assessment of Ki* for ZOL in both the Y204F and Y204A mutants was problematic as the general assumption of a 1:1 enzyme:inhibitor binding ratio as used in all the other Ki* determinations did not appear valid in this case. The estimation of inhibitor concentration required for 50% inhibition (IC_50_) corresponded to a value lower than half of the enzyme concentration, making the determination of Ki* and K_isom_ impossible. The IC_50_ was the only available way of determining the strength of ZOL inhibition (Table 5). The same Y204F and Y204A preparations did not exhibit this behaviour with other inhibitors and identical solutions of ZOL showed normal tight binding with wtFPPS enzyme (Table 3). One possible explanation is that one molecule of ZOL is capable of inhibiting both molecules of FPPS in the enzyme dimer, however we have no corroborating evidence for this theory. The crystal structure of Y204A FPPS in complex with ZOL showed no alterations in the electrostatic interactions and position of key residues in the formation of the allylic binding pocket (Fig. 4B).

### 4.8 Interaction of amino-alkyl BPs with the Thr201 and Tyr204 mutants

In enzyme inhibition experiments, all of the mutants examined with the exception of Y204F showed that the final inhibition of FPPS by PAM, ALN and IBN was narrowly affected by changes to Thr201 or Tyr204. The competitive inhibition of IBN and ALN was increased in both the T201S and T201A enzymes (Table 3). The implication is that the active site of these mutants allows better initial binding of ALN and IBN. However, overall inhibition and K_isom_ are decreased suggesting that binding in the isomerized state is less tight. The results for PAM are mixed with it being a slightly better competitive inhibitor for T201A and somewhat weaker competitive inhibitor for T201S. The structure of wtFPPS with PAM (2 F89) does not offer any insights as to why this would be so since in that structure the PAM nitrogen is within hydrogen-bonding distance of the Tyr204 hydroxyl (3.0 Å) and does not interact directly with Thr201.

Interestingly, the Ki values obtained for both Tyr204 mutants with PAM were similar to the wtFPPS, indicating that, even after disruption of any putative hydrogen bond between PAM and the Tyr residue, the BP is still capable of competing with GPP. Finally, the overall inhibition of Y204F FPPS was significantly increased by ALN and less so by PAM and IBN (p < 0.01). One possible explanation is that the IPP forms a tighter complex, with the amino-alkyl BPs in Y204F. In the wtFPPS ternary structure, the IPP tail is sterically constrained between Asp243 (3.4 Å) and the Tyr204 hydroxyl (3.6 Å). Without the hydroxyl present, the IPP C4 can shift 0.9 Å toward Phe204 and into a more hydrophobic environment, as seen in Y204F-RIS-IPP (Fig. 4C). For PAM, this hydrophobic interaction is lost in the Y204A mutant and is reflected in the return of Ki* to slightly more than wtFPPS (Table 3).

## 5 Dscussion

The availability of crystal structures of FPPS in complex with bisphosphonates identified several active site residues that could play a role in N-BP binding and also in catalysis. Here we use mutants of human FPPS complexed with BPs to elucidate the involvement of Tyr204 and the aliphatic portion of the Thr201 side chain in FPPS substrate binding, in the catalytic mechanism, and in interactions with N-BPs that can influence the strength of drug binding.

Given the high degree of conservation of Thr201 and Tyr204 among different species, along with computer modelling and structure activity relationship studies of BP inhibitors [16], [17], [35], it was postulated that these residues play a pivotal role in allylic substrate binding. Surprisingly, no major alteration in K_m_^GPP^ was observed in all the FPPS mutants examined here even though in the *Escherichia coli* FPPS calculations of the stabilization effect of Thr201 on the carbocation species (1.5 Kcal/mol) suggest a more substantial role of the Thr201 residue in catalysis than the one reported here [35]. Compensation by the other active site residues predicted to stabilize the carbocation intermediate, such as Gln240 and the carbonyl of Lys200, [12], [17] might account for this lack of effect.

Unexpectedly, there was an increased K_m_^IPP^ for all the mutants suggesting a role of the methyl group of Thr201 in the formation of the IPP binding site; however it was the Tyr204 mutants that showed a greater reduction in affinity for IPP. This reduced binding of IPP in both Tyr204 mutants suggests involvement of this residue in the isomerisation event responsible for the formation of the IPP binding site, the orientation of the IPP substrate, or in the stabilization of the carbocation. Our crystal structures of Tyr204 FPPS mutants provide evidence that the Tyr204 hydroxyl forms a hydrogen bond network that correctly orients the IPP-binding residues Gln96 and Arg60 making Tyr204 an essential residue in the second substrate binding event/catalytic process.

In our early experiments we detected the abolition of the acidic pKa value in Tyr204 mutants, a result which indicates the disruption of a possible ionization mechanism [36]. Considering that the pKa value of the tyrosine group is approximately 10, the reduced pKa value of the wtFPPS enzyme might correspond to an interaction of the Tyr204 residue with an adjacent basic residue such as Arg60 or Arg112. These results suggest a possible role of the hydroxyl group of Tyr204 as a proton donor where its removal alters the pH activity profile of the enzyme. If the ionization step in FPPS catalysis progresses via removal of a negative charge from the allylic pyrophosphate [13], formation of a tyrosinate ion, derived from the interaction of the OH group of Tyr with the basic Arg residues (such as Arg112/Arg60) in the vicinity could accelerate the reaction. However the higher activity of the Tyr204 mutants observed at low pH is inconsistent with the proposal that such a tyrosinate ion acts to force IPP into a catalytically competent position, but rather backs up the theory that the stabilization of the PPi leaving group facilitated by the tri-nuclear Mg cluster helps drive the reaction [12]. Chemical rescue experiments of the mutated tyrosine by addition of low molecular weight phenols failed to substitute for the loss of the pKa of a titratable group or to restore the catalytic activity (data not shown). In addition, it is not always possible to attribute a pKa value to a single group, as an ionization state observed might be the outcome of multiple ionizing groups taking part in catalysis [37].

The position of the nitrogen relative to the phosphonate groups in the bisphosphonate is critical for inhibitor potency as we have shown previously [19]. In the case of RIS it was surprising that inhibition was not really affected by the T201A mutation as placement of the nitrogen away from the optimal position on the ring leads to a drop in potency, however it is possible that interaction with the Lys200 carbonyl makes up for the lack of interaction with the Thr201 hydroxyl moiety. In this scenario the nitrogen of RIS needs to be protonated and it is uncertain whether RIS in the active site is protonated [38] at the usual pH at which the crystals are grown (pH 7.5). The T201A mutant structure 4Q23 was crystallised at pH 4.6 and hence should be protonated in this structure. ZOL however is mainly protonated at pH 7.5 and the T201A has a much larger effect on loss of inhibition, which is recovered with the T201S mutant. The results seen with the sterically restricted analogues of RIS, NE58025 1R6S and NE58025 1S6R, also reinforce the importance of the BP side chain nitrogen interactions with Thr201. The inhibition of the Tyr204 mutants by ZOL was of particular interest. The IC_50_ for the inhibition indicates that one molecule of ZOL was inhibiting more than one molecule of the mutant FPPS. It is possible that by binding to one member of the dimer pair, one ZOL molecule prevents both from turning over substrate, maybe by locking the FPPS into a rigid conformation that prevents its twin from moving.

PAM and IBN were not predicted to form any interaction with Thr201 and this is reflected in the lack of change in their inhibition seen in the mutants of this residue. However ALN was predicted to form an H-bond with Thr201 but not the carbonyl of Lys200 [17] and surprisingly there was no change in inhibition with the T201A mutant. It has been suggested that the PAM nitrogen could H-bond to the OH of Tyr204 [17] and it is possible that ALN might also make this interaction. However in the Y204F mutant any expected reduction in inhibition seems to be masked by an unexpected interaction, possibly due to movement of the IPP into a more hydrophobic environment, as the inhibition returns to that of wtFPPS when the ring of this residue is removed and replaced with the methyl in Y204A.

In conclusion, we have identified novel functions of the human FPPS active site residues Thr201 and Tyr204 in catalysis as well as substrate and inhibitor binding. This study provides new insights into the molecular mechanisms of actions of N-BPs by illustrating the involvement of multiple interactions between the side chain of N-BPs with Thr201 and Tyr204 residues, which contribute to their strength of binding. Further understanding of bisphosphonate interaction with FPPS aids in explaining the clinical potency of the bisphosphonates. We hope that this information would also enable the future development of bisphosphonates or other specific FPPS inhibitors for other applications.

## Author contributions

Maria Tsoumpra, Aaron Kwassi, James Dunford and Artem Evdokimov designed and undertook experimental work. Maria Tsoumpra, Joao Muniz, Bobby Barnett, Richard Walters, Frank von Delft, Ewa Pilka, and Kathryn Kavanagh all participated in the crystal mounting, shooting, model building, proof reading and deposition of the X-ray structures. Maria Tsoumpra, Kathryn Kavanagh and James Dunford analysed the data. James Dunford, Frank H. Ebetino, Udo Oppermann and R. Graham Russell were involved in the conceptualisation of the project. The paper was written by Maria Tsoumpra, Kathryn Kavanagh, Frank H. Ebetino, Udo Oppermann, R. Graham Russell and James Dunford.

## Funding

Research was supported through funding from the Oxford NIHR Biomedical Research Unit, Arthritis Research UK (program grant 20522), the Rosetrees Trust, Medical Research Council and BBSRC. The Structural Genomics Consortium is a registered charity (number 1097737) that receives funds from Abbvie, Bayer Healthcare, Boehringer Ingelheim, the Canadian Institutes for Health Research, the Canadian Foundation for Innovation, Eli Lilly and Company, Genome Canada, GlaxoSmithKline, the Ontario Ministry of Economic Development and Innovation, Janssen, the Novartis Research Foundation, Pfizer, Takeda, and the Wellcome Trust [092809/Z/10/Z]. We thank the Alliance for better Bone Health (Procter & Gamble Pharmaceuticals and Sanofi-Aventis) for financial support of this work through an unrestricted educational grant.

## Figures and Tables

**Table 1:**
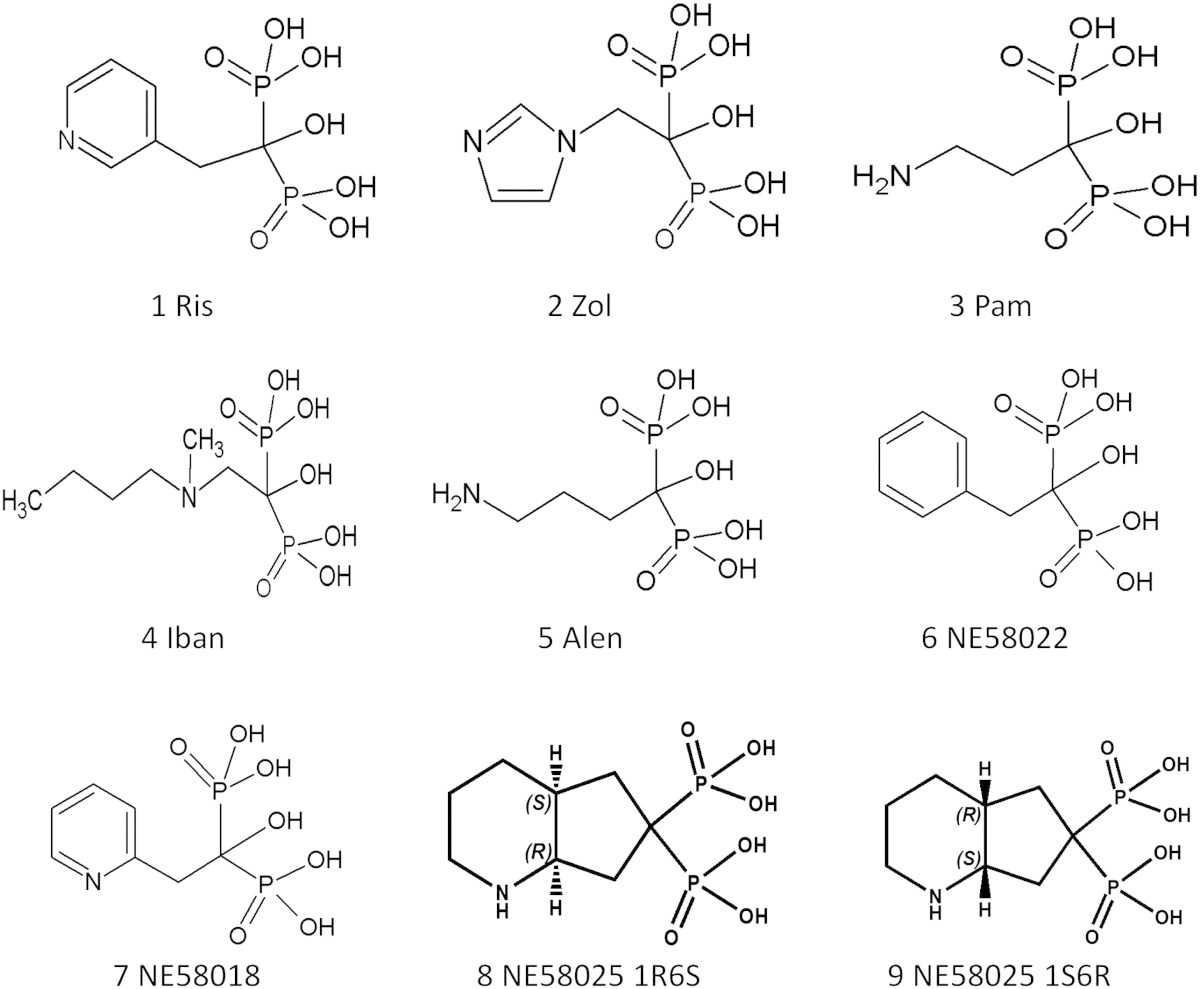
BPs used in the study.

**Table 2:** Kinetic parameters calculated for FPPS wtFPPS and mutants^a^.

	WT	T201S	T201A	Y204F	Y204A
K_m_^GPP^(μM)	2.1 ± 0.2	1.2 ± 0.1	1.5 ± 0.2	1.8 ± 0.6	2.1 ± 0.4
K_m_^IPP^(μM)	1.8 ± 0.3	10.6 ± 1.1	18 ± 2	34 ± 5	44 ± 5
k_cat_(s^− 1^)	0.42	0.24	0.39	0.085	0.052
k_cat_/K_m_^GPP^(μM^− 1^ · s^− 1^)	0.20	0.20	0.26	0.047	0.025
k_cat_/K_m_^IPP^(μM^− 1^ · s^− 1^)	0.23	0.023	0.022	0.0025	0.0012

a Results are expressed as means ± SEM, n ≥ 6, R^2^ ≥ 0.95.

**Table 3:** Estimation of initial inhibition (Ki), isomerised inhibition (Ki*) and K_isom_ of WT, Thr201 and Tyr204 FPPS mutants derived from inhibition assays with N-BPs^a^.

Compound	Construct	Ki (nM)	Ki* (nM)	K_isom_
RIS	WT T201S T201A Y204F Y204A	56.6 ± 3.1 38.7 ± 3.0 61.6 ± 10.8 58.3 ± 4.5 17.7 ± 1.3	0.8 ± 0.06 0.05 ± 0.01 0.16 ± 0.03 2.3 ± 0.4 3.2 ± 0.8	69.7 773 384 24.3 4.5
ZOL	WT T201S T201A Y204F Y204A	62.9 ± 5.1 82.1 ± 4.0 72.7 ± 5.0 54.2 ± 4.4 29.8 ± 3.1	0.06 ± 0.03 0.23 ± 0.1 2.2 ± 0.3 ND ND	1047 355 32.0 ND ND
PAM	WT T201S T201A Y204F Y204A	181.5 ± 6 262.8 ± 27.6 132.0 ± 14.9 199.9 ± 10.4 195.6 ± 9.8	51.9 ± 3.3 43.7 ± 5.7 45.0 ± 5.0 21.7 ± 1.8 73.6 ± 7.7	2.5 5.0 1.9 8.2 1.7
IBN	WT T201S T201A Y204F Y204A	207.7 ± 13.4 48.6 ± 5.3 31.8 ± 3.6 233.1 ± 10.5 142.2 ± 6.0	4.6 ± 0.4 8.1 ± 0.8 5.5 ± 0.6 2.9 ± 0.6 4.3 ± 0.8	44.1 5.0 4.8 79.3 32.1
ALN	WT T201S T201A Y204F Y204A	388.3 ± 31.3 118.1 ± 4.8 191.9 ± 20.2 431.2 ± 37.3 253.9 ± 14.4	56.5 ± 4.9 81.9 ± 7.5 86.5 ± 7.6 7.3 ± 1.0 26.9 ± 2.5	5.9 0.4 1.2 58.1 8.4

ND: not determined. Results are expressed as means ± SEM, R^2^ > 0.95 and n ≥ 6.

a Ki and Ki*and K_isom_ calculated as described.

**Table 4:** Estimation of initial inhibition (Ki), isomerised inhibition (Ki*) and K_isom_ of wtFPPS and T201A FPPS mutants derived from inhibition assays with analogues of RIS^a^.

Analogues of RIS	Enzyme	Ki (nM)	Ki* (nM)	K_isom_
NE58018	WT T201A	58.9 ± 3.6 50.9 ± 5.0	0.7 ± 0.1 2.1 ± 0.2	83.1 23.2
NE58022	WT T201A	365.0 ± 15.9 302.1 ± 17.6	301.0 ± 17.6 302.4 ± 27.9	0.2 0.0
NE58025, 1R6S	WT T201A	710.9 ± 35.8 192.0 ± 11.0	1.7 ± 0.2 13.3 ± 4.0	417 13.4
NE58025, 1S6R	WT T201A	2762 ± 167 1541 ± 237	50.7 ± 7.7 53.5 ± 11.5	53.4 27.8

Results are expressed as means ± SEM, *R*^2^ > 0.95 and n ≥ 4.

a Ki and Ki* and K_isom_ calculated as described in experimental session.

**Table 5:** IC_50_ values of mutant enzymes for final inhibition of ZOL, compared to the wtFPPS inhibition data.

Enzyme	[Enzyme] nM	Theoretical max IC_50_ (nM) ZOL	Experimental IC_50_ZOL (nM)
Y204F	134	67	34 ± 2.2
Y204A	292	146	92.9 ± 5.3
WT	8	4	3.8 ± 0.2

**Table 6:** Summary of crystallographic models.

Enzyme complex	T210S-RIS	T201A-RIS	T201A-ZOL	Y204F-RIS-IPP	Y204A-PAM	Y204A-ZOL	Y204A-RIS-IPP	Y204A-ALN-IPP
PDB accession code	2QIS	4Q23	4KFA	4KPD	4KPJ	4KQ5	4KQS	4KQU
Resolution (Å)	1.80	1.98	1.98	1.96	1.95	1.97	1.97	2.07
R_work_/R_free_ (%)	17.8/20.6	19.2/22.3	18.1/20.1	19.4/22.6	17.2/21.5	17.0/20.4	17.0/20.4	17.6/20.1

## References

[1] Buhaescu I., Izzedine H. (2007). Mevalonate pathway: a review of clinical and therapeutical implications. Clin. Biochem..

[2] Dunford J.E., Thompson K., Coxon F.P., Luckman S.P., Hahn F.M., Poulter C.D., Ebetino F.H., Rogers M.J. (2001). Structure–activity relationships for inhibition of farnesyl diphosphate synthase in vitro and inhibition of bone resorption in vivo by nitrogen-containing bisphosphonates. J. Pharmacol. Exp. Ther..

[3] Rogers M.J., Gordon S., Benford H.L., Coxon F.P., Luckman S.P., Monkkonen J., Frith J.C. (2000). Cellular and molecular mechanisms of action of bisphosphonates. Cancer.

[4] Luckman S.P., Coxon F.P., Ebetino F.H., Russell R.G., Rogers M.J. (1998). Heterocycle-containing bisphosphonates cause apoptosis and inhibit bone resorption by preventing protein prenylation: evidence from structure–activity relationships in J774 macrophages. J. Bone Miner. Res..

[5] Coxon F.P., Helfrich M.H., Van't Hof R., Sebti S., Ralston S.H., Hamilton A., Rogers M.J. (2000). Protein geranylgeranylation is required for osteoclast formation, function, and survival: inhibition by bisphosphonates and GGTI-298. J. Bone Miner. Res..

[6] Ebetino F.H., Hogan A.M., Sun S., Tsoumpra M.K., Duan X., Triffitt J.T., Kwaasi A.A., Dunford J.E., Barnett B.L., Oppermann U., Lundy M.W., Boyde A., Kashemirov B.A., McKenna C.E., Russell R.G. (2011). The relationship between the chemistry and biological activity of the bisphosphonates. Bone.

[7] Reid I.R., Hosking D.J. (2011). Bisphosphonates in Paget's disease. Bone.

[8] Morgan G.J., Davies F.E., Gregory W.M., Cocks K., Bell S.E., Szubert A.J., Navarro-Coy N., Drayson M.T., Owen R.G., Feyler S., Ashcroft A.J., Ross F., Byrne J., Roddie H., Rudin C., Cook G., Jackson G.H., Child J.A. (2010). First-line treatment with zoledronic acid as compared with clodronic acid in multiple myeloma (MRC Myeloma IX): a randomised controlled trial. Lancet.

[9] Coleman R.E., McCloskey E.V. (2011). Bisphosphonates in oncology. Bone.

[10] Russell R.G., Watts N.B., Ebetino F.H., Rogers M.J. (2008). Mechanisms of action of bisphosphonates: similarities and differences and their potential influence on clinical efficacy. Osteoporos. Int..

[11] Eastell R., Walsh J.S., Watts N.B., Siris E. (2011). Bisphosphonates for postmenopausal osteoporosis. Bone.

[12] Hosfield D.J., Zhang Y., Dougan D.R., Broun A., Tari L.W., Swanson R.V., Finn J. (2004). Structural basis for bisphosphonate-mediated inhibition of isoprenoid biosynthesis. J. Biol. Chem..

[13] Poulter C.D., Rilling H.C. (1976). Prenyltransferase: the mechanism of the reaction. Biochemistry.

[14] Poulter C.D., Argyle J.C., Mash E.A. (1978). Farnesyl pyrophosphate synthetase. Mechanistic studies of the 1′-4 coupling reaction with 2-fluorogeranyl pyrophosphate. J. Biol. Chem..

[15] Gabelli S.B., McLellan J.S., Montalvetti A., Oldfield E., Docampo R., Amzel L.M. (2006). Structure and mechanism of the farnesyl diphosphate synthase from Trypanosoma cruzi: implications for drug design. Proteins.

[16] Kavanagh K.L., Guo K., Dunford J.E., Wu X., Knapp S., Ebetino F.H., Rogers M.J., Russell R.G., Oppermann U. (2006). The molecular mechanism of nitrogen-containing bisphosphonates as antiosteoporosis drugs. Proc. Natl. Acad. Sci. U. S. A..

[17] Rondeau J.M., Bitsch F., Bourgier E., Geiser M., Hemmig R., Kroemer M., Lehmann S., Ramage P., Rieffel S., Strauss A., Green J.R., Jahnke W. (2006). Structural basis for the exceptional in vivo efficacy of bisphosphonate drugs. ChemMedChem.

[18] Martin M.B., Arnold W., Heath H.T., Urbina J.A., Oldfield E. (1999). Nitrogen-containing bisphosphonates as carbocation transition state analogs for isoprenoid biosynthesis. Biochem. Biophys. Res. Commun..

[19] Dunford J.E., Kwaasi A.A., Rogers M.J., Barnett B.L., Ebetino F.H., Russell R.G., Oppermann U., Kavanagh K.L. (2008). Structure–activity relationships among the nitrogen containing bisphosphonates in clinical use and other analogues: time-dependent inhibition of human farnesyl pyrophosphate synthase. J. Med. Chem..

[20] Varela I., Pereira S., Ugalde A.P., Navarro C.L., Suarez M.F., Cau P., Cadinanos J., Osorio F.G., Foray N., Cobo J., de Carlos F., Levy N., Freije J.M., Lopez-Otin C. (2008). Combined treatment with statins and aminobisphosphonates extends longevity in a mouse model of human premature aging. Nat. Med..

[21] Russell R.G. (2011). Bisphosphonates: the first 40 years. Bone.

[22] Ling Y., Sahota G., Odeh S., Chan J.M., Araujo F.G., Moreno S.N., Oldfield E. (2005). Bisphosphonate inhibitors of Toxoplasma gondi growth: in vitro, QSAR, and in vivo investigations. J. Med. Chem..

[23] Rodriguez N., Bailey B.N., Martin M.B., Oldfield E., Urbina J.A., Docampo R. (2002). Radical cure of experimental cutaneous leishmaniasis by the bisphosphonate pamidronate. J. Infect. Dis..

[24] Yardley V., Khan A.A., Martin M.B., Slifer T.R., Araujo F.G., Moreno S.N., Docampo R., Croft S.L., Oldfield E. (2002). In vivo activities of farnesyl pyrophosphate synthase inhibitors against Leishmania donovani and Toxoplasma gondii. Antimicrob. Agents Chemother..

[25] Hudock M.P., Sanz-Rodriguez C.E., Song Y., Chan J.M., Zhang Y., Odeh S., Kosztowski T., Leon-Rossell A., Concepcion J.L., Yardley V., Croft S.L., Urbina J.A., Oldfield E. (2006). Inhibition of Trypanosoma cruzi hexokinase by bisphosphonates. J. Med. Chem..

[26] Sanz-Rodriguez C.E., Concepcion J.L., Pekerar S., Oldfield E., Urbina J.A. (2007). Bisphosphonates as inhibitors of Trypanosoma cruzi hexokinase: kinetic and metabolic studies. J. Biol. Chem..

[27] Artz J.D., Wernimont A.K., Dunford J.E., Schapira M., Dong A., Zhao Y., Lew J., Russell R.G., Ebetino F.H., Oppermann U., Hui R. (2010). Molecular characterization of a novel geranylgeranyl pyrophosphate synthase from Plasmodium parasites. J. Biol. Chem..

[28] Ghosh S., Chan J.M., Lea C.R., Meints G.A., Lewis J.C., Tovian Z.S., Flessner R.M., Loftus T.C., Bruchhaus I., Kendrick H., Croft S.L., Kemp R.G., Kobayashi S., Nozaki T., Oldfield E. (2004). Effects of bisphosphonates on the growth of Entamoeba histolytica and Plasmodium species in vitro and in vivo. J. Med. Chem..

[29] Williams J.W., Morrison J.F. (1979). The kinetics of reversible tight-binding inhibition. Methods Enzymol..

[30] Powell H.R. (1999). The Rossmann Fourier autoindexing algorithm in MOSFLM. Acta Crystallogr. D Biol. Crystallogr..

[31] Evans P. (2006). Scaling and assessment of data quality. Acta Crystallogr. D Biol. Crystallogr..

[32] McCoy A.J., Grosse-Kunstleve R.W., Adams P.D., Winn M.D., Storoni L.C., Read R.J. (2007). Phaser crystallographic software. J. Appl. Crystallogr..

[33] Emsley P., Cowtan K. (2004). Coot: model-building tools for molecular graphics. Acta Crystallogr. D Biol. Crystallogr..

[34] McCoy A.J., Grosse-Kunstleve R.W., Storoni L.C., Read R.J. (2005). Likelihood-enhanced fast translation functions. Acta Crystallogr. D Biol. Crystallogr..

[35] Sanchez V.M., Crespo A., Gutkind J.S., Turjanski A.G. (2006). Investigation of the catalytic mechanism of farnesyl pyrophosphate synthase by computer simulation. J. Phys. Chem. B.

[36] Brocklehurst K. (1994). A sound basis for pH-dependent kinetic studies on enzymes. Protein Eng..

[37] Zographos S.E., Oikonomakos N.G., Dixon H.B., Griffin W.G., Johnson L.N., Leonidas D.D. (1995). Sulphate-activated phosphorylase b: the pH-dependence of catalytic activity. Biochem. J..

[38] Hounslow A.M., Carran J., Brown R.J., Rejman D., Blackburn G.M., Watts D.J. (2008). Determination of the microscopic equilibrium dissociation constants for risedronate and its analogues reveals two distinct roles for the nitrogen atom in nitrogen-containing bisphosphonate drugs. J. Med. Chem..

